# Relationship between circulating metabolites and diabetic retinopathy: a two-sample Mendelian randomization analysis

**DOI:** 10.1038/s41598-024-55704-3

**Published:** 2024-02-29

**Authors:** Lingli Ma, Ying Dong, Zimeng Li, Jian Meng, Bingqi Zhao, Qing Wang

**Affiliations:** 1 Department of Endocrinology and Metabolism, China-Japan Union Hospital of Jilin University, 126 Sendai Avenue, Changchun City, Jilin Province China; 2 Department of Radiotherapy, Jilin Cancer Hospital, Changchun, China

**Keywords:** Computational biology and bioinformatics, Genetics, Biomarkers, Endocrinology

## Abstract

Diabetic retinopathy (DR) is the most frequent microvascular complication of diabetes mellitus, however, its underlying biological mechanisms remain poorly understood. We examined single nucleotide polymorphisms linked to 486 blood metabolites through extensive genome-wide association studies conducted on individuals of European ancestry. The FinnGen Biobank database served as a reference to define DR. Two-sample MR analysis was conducted to reveal the association between the levels of genetically predicted circulating metabolites and the susceptibility to DR. To validate the robustness of the obtained findings, sensitivity analyses with weighted median, weighted mode, and MR-Egger were conducted. 1-oleoylglycerophosphoethanolamine (odds ratio [OR] (OR per one standard deviation [SD] increase) = 0.414; 95% confidence interval [CI] 0.292–0.587; *P* = 7.613E−07, P_FDR_ = 6.849E−06), pyroglutamine (OR per one SD increase = 0.414; 95% confidence interval [CI] 0.292–0.587; *P* = 8.31E−04, P_FDR_ = 0.007), phenyllactate (PLA) (OR per one SD increase = 0.591; 95% confidence interval [CI] 0.418–0.836; *P* = 0.003, P_FDR_ = 0.026), metoprolol acid metabolite (OR per one SD increase = 0.978; 95% confidence interval [CI] 0.962–0.993; *P* = 0.005, P_FDR_ = 0.042), 10-undecenoate (OR per one SD increase = 0.788; 95% confidence interval [CI] 0.667–0.932; *P* = 0.005, P_FDR_ = 0.049), erythritol (OR per one SD increase = 0.691; 95% confidence interval [CI] 0.513–0.932; *P* = 0.015, P_FDR_ = 0.034), 1-stearoylglycerophosphoethanolamine (OR per one SD increase = 0.636; 95% confidence interval [CI] 0.431–0.937; *P* = 0.022, P_FDR_ = 0.099), 1-arachidonoylglycerophosphoethanolamine (OR per one SD increase = 0.636; 95% confidence interval [CI] 0.431–0.937; *P* = 0.030, P_FDR_ = 0.099) showed a significant causal relationship with DR and could have protective effects. stachydrine (OR per one SD increase = 1.146; 95% confidence interval [CI] 1.066–1.233; *P* = 2.270E−04, P_FDR_ = 0.002), butyrylcarnitine (OR per one SD increase = 1.117; 95% confidence interval [CI] 1.023–1.219; *P* = 0.014, P_FDR_ = 0.062), 5-oxoproline (OR per one SD increase = 1.569; 95% confidence interval [CI] 1.056–2.335; *P* = 0.026, P_FDR_ = 0.082), and kynurenine (OR = 1.623; 95% CI 1.042–2.526; *P* = 0.041, P_FDR_ = 0.097) were significantly associated with an increased risk of DR. This study identified metabolites have the potential to be considered prospective compounds for investigating the underlying mechanisms of DR and for selecting appropriate drug targets.

## Introduction

Diabetic retinopathy (DR) is the most common microvascular complication of diabetes and the leading cause of preventable blindness in the adult working population^1,2^. According to the Global Burden of Disease study, DR ranks fifth as the most common cause of blindness and substantial visual impairment in individuals aged 50 years and older^3^. Based on projections, the global count of individuals afflicted by DR is expected to increase to 129.84 million by 2030, with an estimated surge to 160.5 million by 2045^1^.

DR is characterized by neurovascular degeneration due to chronic hyperglycemia. Proliferative diabetic retinopathy (PDR) is a severe complication of DR, with a risk of progression to complete loss of both central and peripheral vision^4^. The global prevalence of DR among diabetic patients has reached 34.6%. The incidence of PDR, diabetic macular edema, and vision-threatening DR are 7.0%, 6.8%, and 10.2%, respectively^5^. Moreover, the prevalence of DR in prediabetic patients is 6.6% (with a quartile range of 1.9–9.8%), suggesting that there is a long asymptomatic period before DR diagnosis^6^; hence, it is critical to perform frequent retinal examinations in all diabetic patients. Regular retinal screening is an effective approach to prevent DR-related complications. Although significant developments have been made in the recognition and management of DR in recent decades, there is a lack of optimal diagnostic indicators and therapeutic approaches. Furthermore, meeting the demands of eye screening and eye health services for diabetic patients remains a challenging task^7^; therefore, the early identification of prognostic factors that indicate the risk of vision loss is essential to prevent DR progression and reduce the incidence of vision loss.

In recent years, several studies have used the combination of metabolomics and genomics to explore potential markers and reveal the underlying mechanisms of diabetes-related complications, thus broadening the understanding of the pathogenesis of microvascular complications in diabetes^8–11^. Metabolomics is an innovative and efficient analytical method that can comprehensively determine the levels of small metabolites (< 1500 Da)^12^. Because the metabolome is located downstream of the genome, transcriptome, and proteome, it provides key information regarding the dynamics of intricate biological systems. Consequently, metabolomics can facilitate a thorough integrated depiction of the biology of an organism^13,14^. The revolutionary sequencing of the human genome has brought a new era of personalized healthcare, where genetic variations can be used to predict the effects of a particular therapeutic approach for optimizing disease treatment in an individual^13^.

Many previous studies have examined the association between specific metabolites and diseases through metabolomics; however, an apparent limitation of these studies is the inability to determine whether these differences in metabolites or endogenous insulin loss and the subsequent metabolic disturbances contribute to the underlying disease etiology. Mendelian randomization (MR) analysis is an effective technique that utilizes genetic variation as an unconfounded instrumental variable to investigate causal relationships between exposures and outcomes^15,16^. Considering the random allocation of genetic diversity during conception, MR shows less vulnerability to confounding bias and has the potential to establish reverse causation as compared to observational studies^17^, and it has been described as a “naturally occurring randomized double-blind trial.”

Recently, the scope of genome-wide association study (GWAS) has been expanded to include metabolic profiling. This has led to the development of metabolic profiles for genetically determined metabolites (GDMs)^18^. Based on this context, in the present study, a two-sample MR approach was used to analyze the causal impact of human serum metabolites on DR. The study also aimed to identify any common metabolites that may have a potential causal impact on DR. Lastly, we attempted to reveal the metabolic pathways that could potentially contribute to the development of DR.

## Methods and materials

### Study design

**Figure 1:**
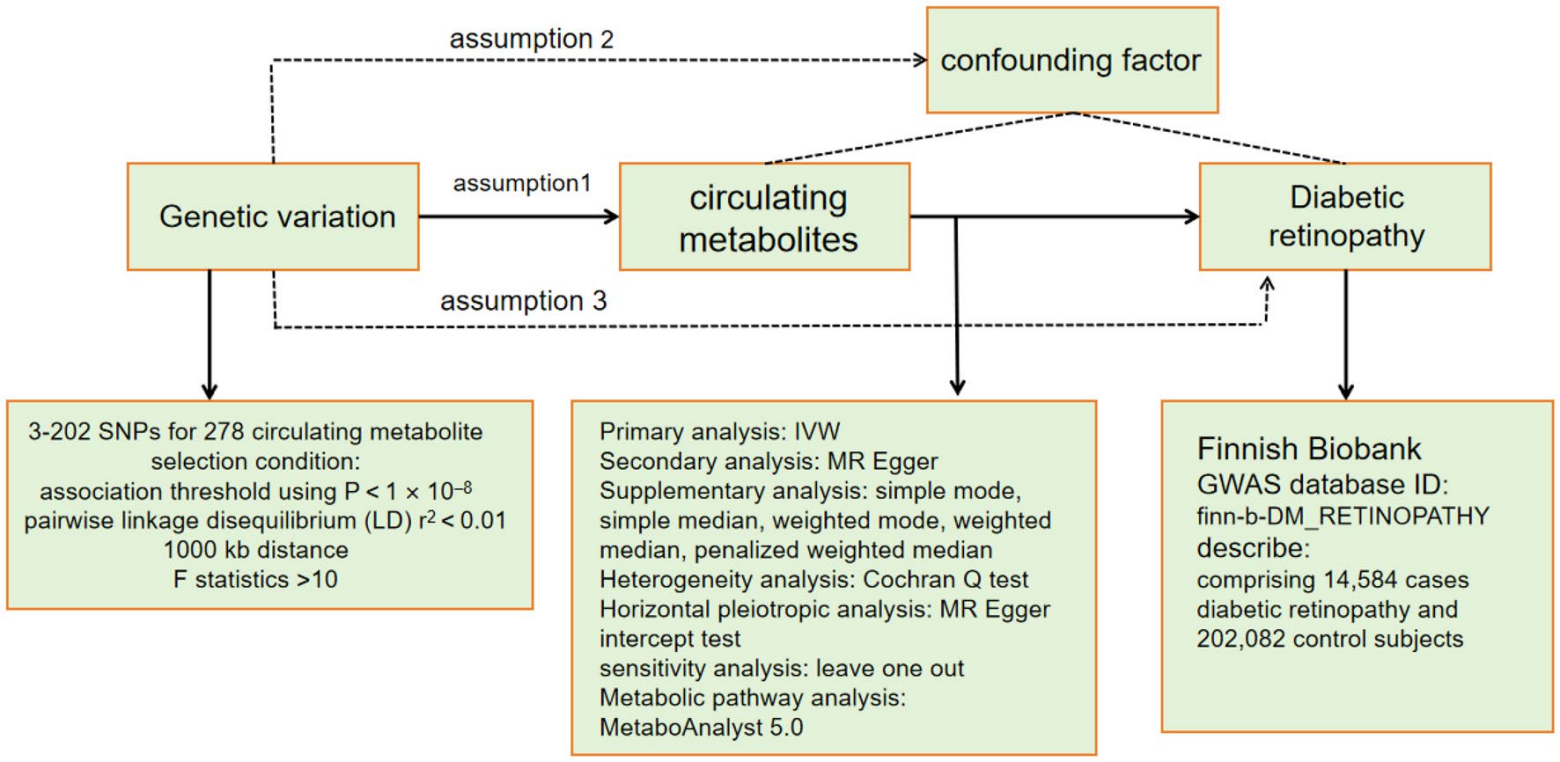
Mendelian randomization model of circulating metabolites on DR. The overall design and abstract of the results of this study.

A two-sample MR design was used to thoroughly evaluate the causal relationship between metabolites detected in the blood of individuals and the probability of developing DR. To effectively design an MR study, the following three assumptions should be met: (1) the genetic instruments exhibit a robust association with the exposure; (2) the genetic instruments are not linked with potential confounding factors; and (3) the genetic instruments exclusively affect the outcome through the specific exposure of interest. The second and third assumptions, commonly referred to as the independence of horizontal pleiotropy, can be evaluated by various statistical methods. We obtained the genetic data for DR from two independent GWAS consortia for the primary analysis (UK Biobank: IEU analysis of UK Biobank phenotypes) and replication analysis (Finnish Biobank: FinnGen biobank analysis round 5). Figure 1 shows an overview of the study design. Statistical analyses were conducted using the “TwoSampleMR” package (version 0.5.7) in the R program (version 3.4.2) and the Review Manager software (version 5.4.1).

### GWAS data for human blood metabolites

The genetic information of each blood metabolite was extracted from the Metabolomics GWAS server (http://metabolomics.helmholtz-muenchen.de/gwas/). Specifically, we collected data on genetic variations from the GWAS conducted by Shin et al.^18^, which involved high-throughput metabolic profiling. A total of 7824 individuals of European descent were registered, and approximately 2.1 million single nucleotide polymorphisms (SNPs) were screened for 486 metabolites. Among these 486 metabolites, 208 remain unidentified because of the lack of conclusive evidence regarding their chemical nature. The remaining 278 metabolites were successfully recognized and classified into eight metabolic groups based on the Kyoto Encyclopedia of Genes and Genomes (KEGG) database. These groups include amino acids, cofactors and vitamins, energy, carbohydrates, lipids, peptides, nucleotides, and xenobiotics.

### GWAS data for DR

The GWAS statistics for DR were obtained from the FinnGen Biobank (GWASID: finn-b-DM_RETINOPATHY; https://gwas.mrcieu.ac.uk/datasets/finn-b-DM_RETINOPATHY/), which comprised 14,584 DR patients and 202,082 control subjects.

### Instrument selection

We performed several procedures to select genetic variants linked to metabolites. Initially, based on the limited number of SNPs attaining genome-wide significance, we fine-tuned the association threshold to *P* < 1 × 10^−6^. We also applied pairwise linkage disequilibrium (r^2^ < 0.001) within a 1000 kb range to identify top independent SNPs. This widely applied method has been used in previous MR studies. Simultaneously, we calculated F statistics for each SNP to assess its statistical strength^19^, thus avoiding bias caused by weak instruments. To ensure that all SNPs contributed ample variance to the corresponding metabolites, we excluded weak instruments with F < 10. We also excluded missing SNPs or those SNPs for which suitable proxies were not identified. Next, we performed harmonization to align the alleles of exposure-SNPs and outcome-SNPs. To maintain dataset consistency, SNPs with palindromic properties or with intermediate effect allele frequencies as well as those with incompatible alleles (e.g., A/G vs. A/C) were eliminated. Finally, we preserved the metabolites that had at least three SNPs for the MR analysis.

### MR analysis

The random-effect inverse-variance weighted (IVW) method, a popular technique used in MR studies, was employed to detect meaningful cause-and-effect relationships between metabolites and DR, with a significance level of *P* < 0.05. IVW merges Wald ratios for every SNP to yield an overall estimation. Specifically, IVW assumes that all genetic variants are genuine, thus rendering it the most robust approach for MR estimation, albeit vulnerable to pleiotropic bias. In the present study, we utilized IVW as the primary approach to initially explore associations between metabolites and DR. We also used seven other MR analysis methods to examine the findings. These methods included the simple mode, simple median, weighted mode, weighted median, penalized weighted median, MR Egger, and MR Egger (bootstrap). These MR analysis methods were used for the sensitivity analysis of our findings.

### Statistical analysis

Statistical analyses were conducted using the “Twosample MR” package in R software version 0.5.7 (R Foundation for Statistical Computing, Vienna, Austria). Based on the 9 MR methods mentioned above, we took the IVW results as the primary MR estimates and considered the consistency of the results across other MR methods. Here, to address multiple hypothesis testing, we estimated the false discovery rate (FDR) adjusted p values (q values), in the main IVW MR analyses, using the sequential p value approach proposed by Benjamini and Hochberg. A q value not greater than 10% was considered significant. Finally, a leave-one-out analysis (LOO) was performed to detect if there is any single SNP disproportionately responsible for the result of each MR study. The effect estimates reflect the increase in DR risk per SD higher in the natural scale of each metabolite. All tests were two-sided, and the analysis was conducted using the “TwoSampleMR” package (packages in R software (version 4.0.2).

### Visualization of results and metabolic pathway analysis

The final results were visualized using the volcanic plot. Forest plots were used for circulating metabolites showing a significant causal association. KEGG pathway enrichment analyses were performed for circulating metabolites with significant differences. MetaboAnalyst 5.0 (http://www.metaboanalyst.ca/) was used to examine the metabolic pathway of the significant circulating metabolites. This database integrates biological data and analytical methods, thereby enabling the systematic annotation of biological functions for extensive lists of genes or proteins. Results were considered statistically significant for *P* < 0.05 and potentially statistically significant for 0.05 < *P* < 0.10.

### Ethics approval and consent to participate

Only publicly available GWAS data were used in this study, and the ethical approval and consent to participate data are available for the original GWAS study.

## Results

Table 1 shows the source of the MR data for the present study. After choosing the appropriate instruments, 278 of 486 metabolites were selected for MR estimation; the remaining 208 metabolites were unidentified (Additional file 1: Table S1: Summary of calculation results of causal effect value estimates by various MR Methods; Table S2: Summary of the results of heterogeneity analysis by various MR Methods; Table S3: Summary of the results of pleiotropy calculations for various MR Methods; Table S4: Raw data summary; Table S5. Summary of calculation results of detailed causal effect value estimates by various MR Methods for significant metabolites). The number of SNPs for each metabolite ranged from 2 to 202. A noteworthy finding was that all F statistics for the SNPs were above 10, thus indicating no usage of weak instruments.

**Table 1:** Summary of genome-wide association studies included in this study.

Phenotype	GWAS data source	Cohort(s)	Sample size	Race	Website
Human blood metabolites	Shin et al., 2014	2 European population studies	7824	European	https://gwas.mrcieu.ac.uk/datasets/
Diabetic retinopathy	finn-b-DM_RETINOPATHY	FinnGen	14,584 cases 202,082 ncontrols	European	https://gwas.mrcieu.ac.uk/datasets/finn-b-DM_RETINOPATHY/

### Estimation of the causal effect of circulating metabolites on DR

**Figure 2:**
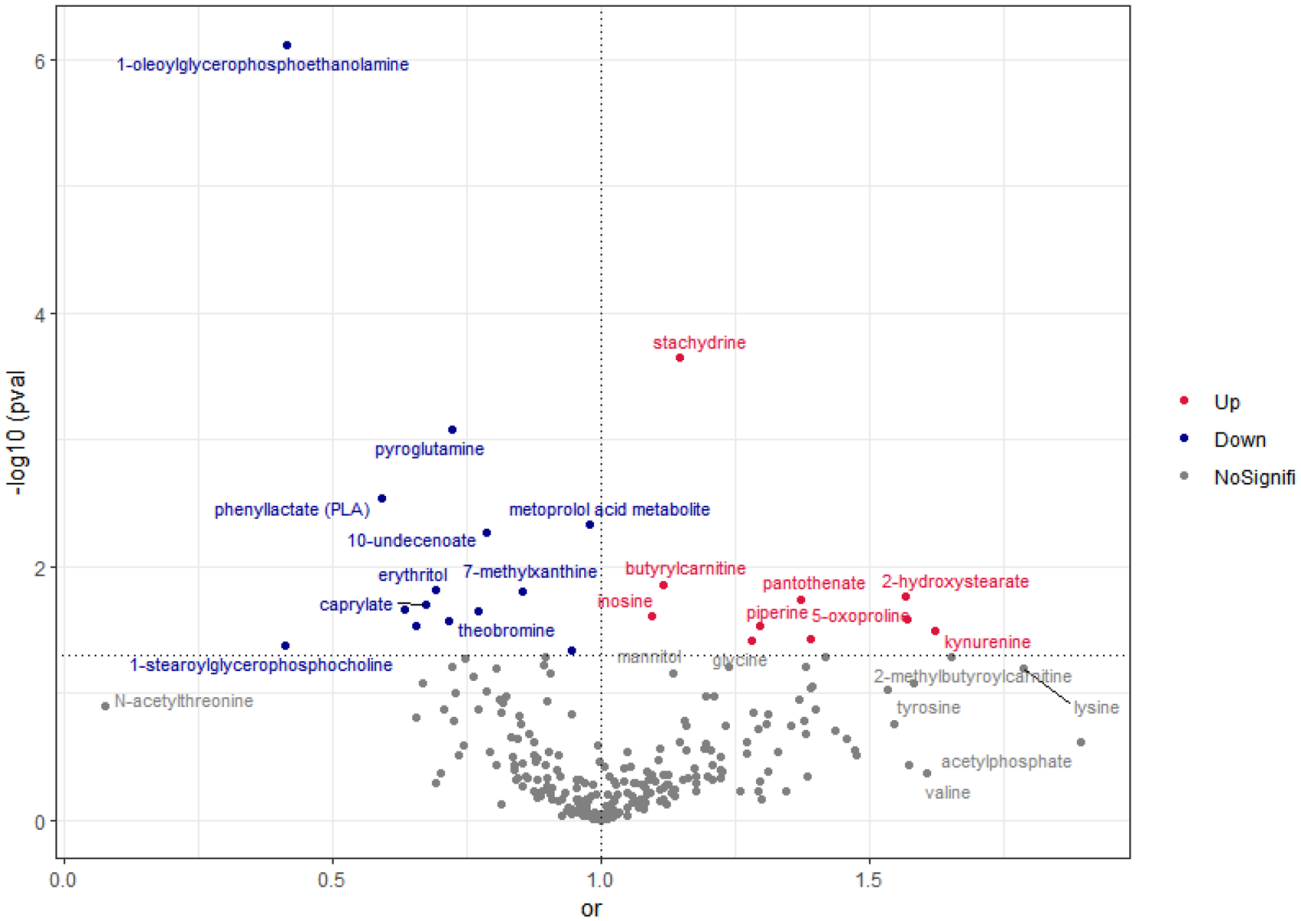
Volcano plot for demonstrating the causal effect estimation and statistical effect estimation of 278 circulating metabolites on DR. IVW method MR results of screening of 278 metabolites as exposures on DR (Sample one). Each point represents a circulating metabolite. The horizontal X-axis is taken as the axis of OR value, OR value greater than 1 and statistically significant is the risk factor, represented by the red dot; OR value less than 1 and statistically significant is the protection factor, represented by the blue dot; serum metabolites with no statistical difference are represented by the gray dot.

Based on MR analysis of the selected 278 metabolites, IVW detected 24 metabolites that showed a significant association with DR (Fig. 2, Table S5). However, after multiple correction tests for FDR, only 12 indicators were significantly correlated with DR, as 1-oleoylglycerophosphoethanolamine (odds ratio [OR] (OR per one standard deviation [SD] increase) = 0.414; 95% confidence interval [CI]: 0.292–0.587; *P* = 7.613E−07, P_FDR_ = 6.849E−06), pyroglutamine (OR per one SD increase = 0.414; 95% confidence interval [CI]: 0.292–0.587; *P* = 8.31E−04, P_FDR_ = 0.007), phenyllactate (PLA) (OR per one SD increase = 0.591; 95% confidence interval [CI]: 0.418–0.836; *P* = 0.003, P_FDR_ = 0.026), metoprolol acid metabolite (OR per one SD increase = 0.978; 95% confidence interval [CI]: 0.962–0.993; *P* = 0.005, P_FDR_ = 0.042), 10-undecenoate (OR per one SD increase = 0.788; 95% confidence interval [CI]: 0.667–0.932; *P* = 0.005, P_FDR_ = 0.049), erythritol (OR per one SD increase = 0.691; 95% confidence interval [CI]: 0.513–0.932; *P* = 0.015, P_FDR_ = 0.034), 1-stearoylglycerophosphoethanolamine (OR per one SD increase = 0.636; 95% confidence interval [CI]: 0.431–0.937; *P* = 0.022, P_FDR_ = 0.099), 1-arachidonoylglycerophosphoethanolamine (OR per one SD increase = 0.636; 95% confidence interval [CI]: 0.431–0.937; *P* = 0.030, P_FDR_ = 0.099) showed a significant causal relationship with DR and could have protective effects. stachydrine (OR per one SD increase = 1.146; 95% confidence interval [CI]: 1.066–1.233; *P* = 2.270E−04, P_FDR_ = 0.002), butyrylcarnitine (OR per one SD increase = 1.117; 95% confidence interval [CI]: 1.023–1.219; *P* = 0.014, P_FDR_ = 0.062), 5-oxoproline (OR per one SD increase = 1.569; 95% confidence interval [CI]: 1.056–2.335; *P* = 0.026, P_FDR_ = 0.082), and kynurenine (OR = 1.623; 95% CI: 1.042–2.526; *P* = 0.041, P_FDR_ = 0.097) were significantly associated with an increased risk of DR. The detailed results are shown in Table 2. More information as showed in Supplementary Table 1.

**Table 2:** Summary of 278 metabolites have significant causal effect on diabetic retinopathy.

Phenotype	N SNP	OR	95% CI	*P*	P_FDR_
1-oleoylglycerophosphoethanolamine	10	0.414	0.292–0.587	7.61E−07	6.849E−06
Phenyllactate (PLA)	20	0.591	0.418–0.836	0.003	0.026
1-stearoylglycerophosphoethanolamine	14	0.636	0.431–0.937	0.022	0.099
1-arachidonoylglycerophosphoethanolamine	28	0.656	0.450–0.958	0.029	0.099
Erythritol	27	0.691	0.513–0.932	0.015	0.042
Pyroglutamine	20	0.725	0.600–0.875	0.001	0.007
10-undecenoate	30	0.788	0.667–0.932	0.005	0.042
Metoprolol acid metabolite	34	0.978	0.962–0.993	0.005	0.042
Butyrylcarnitine	51	1.117	1.023–1.219	0.014	0.062
Stachydrine	7	1.146	1.066–1.233	2.269E−04	0.002
5-oxoproline	25	1.570	1.056–2.335	0.026	0.082
Kynurenine	44	1.623	1.042–2.526	0.032	0.097

### Heterogeneity analysis and horizontal pleiotropy test for the effects of circulating metabolites on DR

Heterogeneity analysis was conducted for the above mentioned 12 circulating metabolites showing a significant association with DR. The results revealed the presence of heterogeneity for 1-stearoylglycerophosphocholine and kynurenine (Pivw for Cochran Q test: 5.76E−06 and 0.017, P_MR Egger_ for Cochran Q test: 0.092 and 0.016, respectively). No heterogeneity was found for the other circulating metabolites. The detailed results are shown in Supplementary Table 2. Pleiotropy analysis revealed a low risk of horizontal pleiotropy for 1-stearoylglycerophosphocholine, benzoate, catechol sulfate, gamma-glutamylleucine, salicyluric acid, pyruvate, decanoylcarnitine, 1-linoleoylglycerophosphocholine, and asparagine (P_MR Egger_ for intercept test: 0.001, 0.011, 0.011, 0.025, 0.026, 0.033, 0.040, 0.040, and 0.043, respectively). Moreover, the causal effects of the other circulating metabolites on DR were not confounded by horizontal pleiotropy of SNPs. The detailed results are shown in Supplementary Table 3. Furthermore, the results of the LOO analysis confirmed the absence of biased MR estimation for individual SNP (Supplementary Figs. 1–12).

### Metabolic pathway analysis

**Figure 3:**
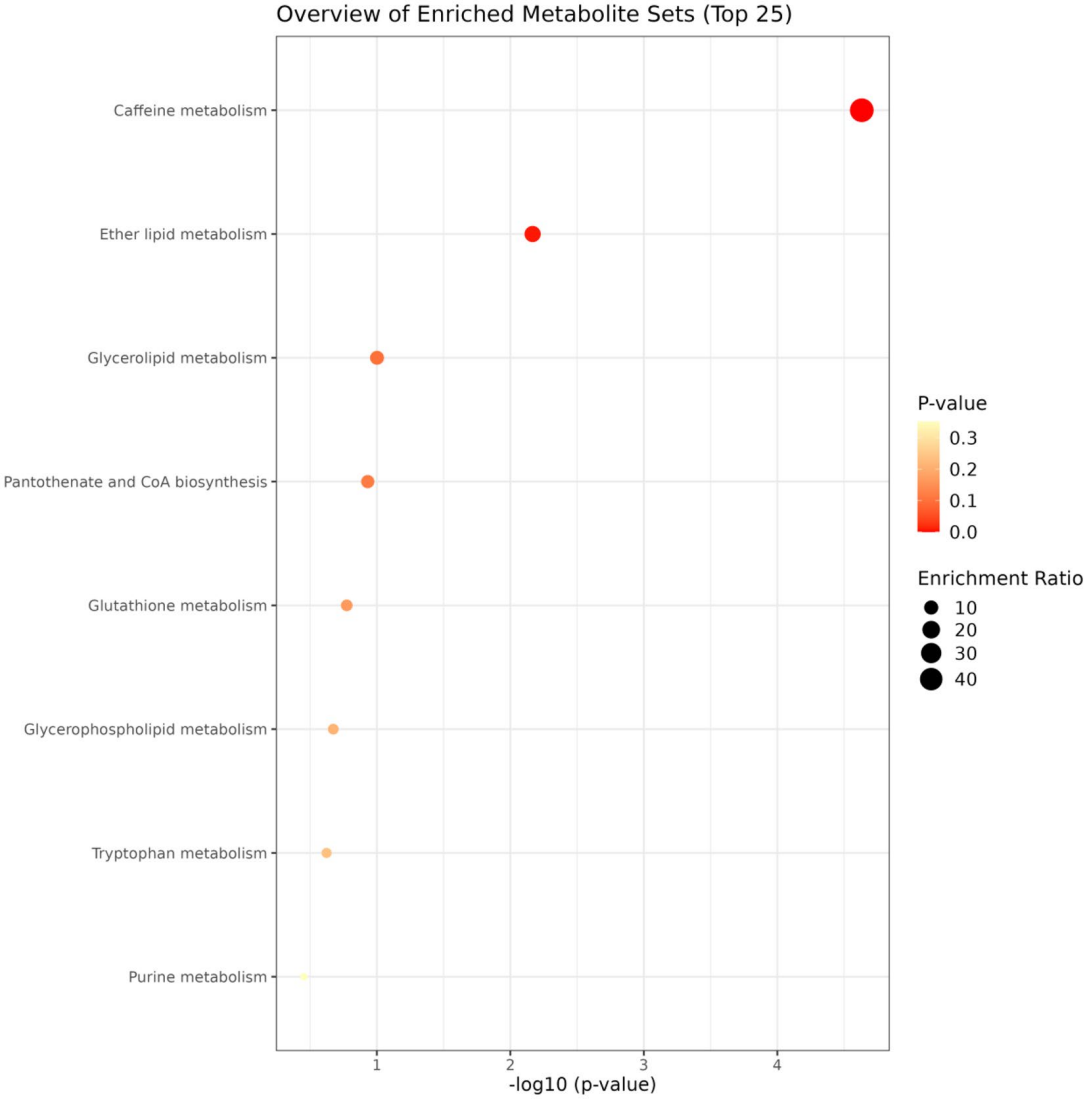
The enrichment analysis for the SNPs of DR. We selected SNPS with higher enrichment rankings.

In the KEGG pathway enrichment analysis, 24 circulating metabolites were assessed for significant variations, and 8 potential metabolic pathways associated with DR were identified. Among these pathways, the three most significant ones were “Caffeine metabolism,” “Ether lipid metabolism,” and “Glycerolipid metabolism” (*P* = 2.33E−5, 0.007, and 0.099, respectively; all *P* < 0.10). The fourth significant pathway was “Pantothenate and CoA biosynthesis,” which also played a crucial role as shown in Fig. 3.

## Discussion

In the present study, we used GWAS data and identified 12 of 486 blood metabolites associated with DR. Of these 12 blood metabolites, 5 showed adverse effects and were associated with an increased risk of DR, including kynurenine, 5-oxoproline, stachydrine, and butyrylcarnitine, while 7 metabolites exhibited protective effects against DR, including 1-oleoylglycerophosphoethanolamine, 1-stearoylglycerophosphocholine, pyroglutamine, phenyllactate (PLA), metoprolol acid metabolite, 10-undecenoate, erythritol, 1-stearoylglycerophosphoethanolamine, 1-arachidonoylglycerophosphoethanolamine. Furthermore, we conducted KEGG pathway enrichment analysis and identified eight metabolic pathways that were significantly associated with DR. The present study is the first to combine metabolomics and genomics analyses to investigate the causal relationship between serum metabolites and DR. The findings provided novel insights into the role of gene-environment interactions in the development of DR and could guide future precision therapy approaches.

Various biofluids such as blood (serum and plasma) and ocular fluids have been used in human studies for multi-omics analysis^20^. A major advantage of using blood is that it provides a global metabolomic profile, thus offering a comprehensive overview of the metabolites present in the body^21^. These studies investigated samples from patients who were diagnosed with either type I diabetes (T1D) or type 2 diabetes (T2D) and showed varying disease duration and stages of DR^9,22–24^. An in vitro study conducted using a fusion of human retinal pigment epithelial cells (ARPE-19) revealed that inducing endoplasmic reticulum stress or depriving the cells of essential nutrients such as specific amino acids (e.g., tryptophan [Trp] or glutamine) notably increased VEGF expression. This finding implies that amino acid metabolism plays a crucial role in the response of the cells to a hypoxic environment^25^. Trp is an essential amino acid that plays a critical role in protein biosynthesis and affects various pathophysiological processes, including neuronal function, metabolism, inflammatory response, oxidative stress, immune response, and intestinal homeostasis^26^. Trp is metabolized to kynurenine by indoleamine 2,2-dioxygenase (IDO). A previous study reported that patients with nonproliferative diabetic retinopathy showed elevated expression levels of IDO and kynurenine, while patients with PDR showed higher levels of kynurenine and no significant change in Trp levels, thus suggesting a possible correlation among IDO, Trp, and DR^27^. A targeted metabolomics study demonstrated that total Dimethyarginine, Trp, and kynurenine were potential indicators of DR progression in patients with T2D^28^, which was consistent with the findings of our present study. Previous studies have demonstrated the role of kynurenine in the development of diabetes mellitus (DM)^29^, and in vitro and in vivo experiments confirmed that kynurenine directly affects glucose metabolism. Kynurenine acts as a systemic integrator of energy metabolism through its effects on adipocytes, immune cells, and muscle cells^30^; it may also inhibit pancreatic insulin secretion and induce apoptosis of pancreatic β-cells through a cysteoaspartate lyase-3-dependent mechanism^31^. In contrast, another study showed that kynurenine increased glucose-induced insulin secretion in pancreatic islets of healthy rats^32^. Untargeted metabolomics revealed a decrease in serum kynurenine levels in diabetic dogs^33^, while other studies have shown no differences in kynurenine levels between T1D patients and controls^34^. Based on the current evidence and the results of our present research, kynurenine could serve as a significant biomarker in blood circulation and a crucial target for treating DR.

The present MR analysis also discovered specific metabolites, some of which have been reported in earlier investigations. Pantothenic acid (Pan), also known as vitamin B5, synthesizes coenzyme A in conjunction with cysteine and ATP; CoA is involved in 4% of all known enzymatic reactions in the body^35^. Pan and CoA biogenesis pathways have critical roles in various cellular physiological and pathological processes^36^, and alterations in the levels of Pan and CoA balance mitochondrial energy metabolism^37^, which is closely associated with DR progression. The present study revealed an increased level of pantothenate as a potential risk factor for DR. In a previous prospective study, 45 patients with T2DM showed blood metabolites different from those observed in 15 control subjects. Furthermore, pathway enrichment analysis indicated that alterations in amino acid metabolism, fatty acid metabolism, and Pan and CoA biosynthesis were associated with the development of DR^38^; moreover, aspartate, glutamine, and Pan were the key factors related to the differential enrichment of these pathways, and Pan level in the vitreous cavity showed an increasing trend. A subgroup analysis of an untargeted study on lipidomics and metabolomics revealed a specific plasma metabolomic profile of DR. This profile exclusively included P-octopamine, Pan, deoxyguanosine monophosphate, and methylglutarylcarnitine as specific markers for DR^39^. Most previous studies suggest that the elevated level of Pan has a protective effect against diabetes and its complications^40^, and that PA regulates CoA synthesis in cell membranes and prevents endothelial dysfunction caused by enhanced oxidative stress^36^. The elevated Pan level might impair CoA biosynthesis, thereby promoting DR progression.

Our present study also has some limitations. First, given the categorization of the raw data, we could not grade the severity of DR and could only analyze DR as a whole entity. Second, because data of only individuals of Finnish descent were analyzed, this limitation hinders the transferability of our results across other ethnic groups; hence, future studies should confirm the generalizability of our findings by validating the data on metabolites detected in populations other than European subjects. Third, this MR study was based on the analysis of the blood metabolome. Although blood is considered a good sample source for metabolite data, some blood metabolites cannot penetrate the blood-retinal barrier, and conducting additional investigations to determine alterations in the metabolites present in the vitreous cavity and atrial fluid can enable to discover more favorable biomarkers and targets for DR therapy. Fourth, although this study recognized various metabolites that contribute to the risk of DR development, additional research is required to reveal their role in the pathogenic mechanisms of DR.

In conclusion, our study identified 12 metabolites that possibly had a causal association with the pathogenesis of DR. Among these metabolites, kynurenine exhibited a potent effect on DR and could serve as a potential therapeutic target for DR. This study also identified several important metabolic pathways that might be relevant to DR pathology. These metabolites and their associated pathways may have utility in clinical settings for the early detection and prevention of DR. They can also be considered as potential molecules for future investigations on the underlying mechanisms and selection of drug targets for DR.

### Supplementary Information

Supplementary Figures.

Supplementary Tables.

## Data Availability

All data generated during this study are included in this published article and the supplementary materials. GWAS summary statistics for human blood metabolites are publicly available at http://metabolomics.helmholtz-muenchen.de/gwas/. GWAS summary statistics for DR from the ILAE consortium and FinnGen consortium are publicly available at https://gwas.mrcieu.ac.uk/datasets/finn-b-DM_NEPHROPATHY/.

## Abbreviations

DR: Diabetic retinopathy
MR: Mendelian randomization
PDR: Proliferative diabetic retinopathy
GWAS: Genome-wide association study
GDMs: Genetically determined metabolites
SNPs: Single nucleotide polymorphisms
KEGG: Kyoto Encyclopedia of Genes and Genomes
IVW: Inverse-variance weighted
OR: Odds ratio
CI: Confidence interval
T1D: Type 1 diabetes
T2D: Type 2 diabetes
Trp: Tryptophan
IDO: Indoleamine 2,2-dioxygenase
DM: Diabetes mellitus
Pan: Pantothenic acid
HFD: High-fat diet
