# Supplementary material for: Relationship between circulating metabolites and diabetic retinopathy: a two-sample Mendelian randomization analysis
Source: Sci Rep. 2024 Feb 29;14:4964. doi: 10.1038/s41598-024-55704-3 (PMC10904376; doi:10.1038/s41598-024-55704-3)
Supplement: Supplementary file 1 — Supplementary Figures. [file 41598_2024_55704_MOESM1_ESM.docx]

**Supplementary Figures**

**Supplementary Figure 1.** Mendelian randomization analyses of effects of 1-oleoylglycerophosphoethanolamine on DR. A, Scatter plot; B, Forest plot ; C, Leave-one-out plot; and D, Funnel plot.

**Supplementary Figure 2.** Mendelian randomization analyses of effects of phenyllactate (PLA) on DR. A, Scatter plot; B, Forest plot ; C, Leave-one-out plot; and D, Funnel plot.

**Supplementary Figure 3.** Mendelian randomization analyses of effects of 1-stearoylglycerophosphoethanolamine on DR. A, Scatter plot; B, Forest plot ; C, Leave-one-out plot; and D, Funnel plot.

**Supplementary Figure 4.** Mendelian randomization analyses of effects of 1-arachidonoylglycerophosphoethanolamine on DR. A, Scatter plot; B, Forest plot ; C, Leave-one-out plot; and D, Funnel plot.

**Supplementary Figure 5.** Mendelian randomization analyses of effects of erythritol on DR. A, Scatter plot; B, Forest plot ; C, Leave-one-out plot; and D, Funnel plot.

**Supplementary Figure 6.** Mendelian randomization analyses of effects of pyroglutamine on DR. A, Scatter plot; B, Forest plot ; C, Leave-one-out plot; and D, Funnel plot.

**Supplementary Figure 7.** Mendelian randomization analyses of effects of 10-undecenoate on DR. A, Scatter plot; B, Forest plot ; C, Leave-one-out plot; and D, Funnel plot.

**Supplementary Figure 8.** Mendelian randomization analyses of effects of metoprolol acid metabolite on DR. A, Scatter plot; B, Forest plot ; C, Leave-one-out plot; and D, Funnel plot.

**Supplementary Figure 9.** Mendelian randomization analyses of effects of butyrylcarnitine on DR. A, Scatter plot; B, Forest plot ; C, Leave-one-out plot; and D, Funnel plot.

**Supplementary Figure 10.** Mendelian randomization analyses of effects of stachydrine on DR. A, Scatter plot; B, Forest plot ; C, Leave-one-out plot; and D, Funnel plot.

**Supplementary Figure 11.** Mendelian randomization analyses of effects of 5-oxoproline on DR. A, Scatter plot; B, Forest plot ; C, Leave-one-out plot; and D, Funnel plot.

**Supplementary Figure 12.** Mendelian randomization analyses of effects of kynurenine on DR. A, Scatter plot; B, Forest plot ; C, Leave-one-out plot; and D, Funnel plot.
